# An Altered Sphingolipid Profile as a Risk Factor for Progressive Neurodegeneration in Long-Chain 3-Hydroxyacyl-CoA Deficiency (LCHADD)

**DOI:** 10.3390/ijms23137144

**Published:** 2022-06-27

**Authors:** Sara Tucci

**Affiliations:** 1 Pharmacy, Medical Center, University of Freiburg, 79106 Freiburg, Germany; sara.tucci@uniklinik-freiburg.de; 2 Department of General Pediatrics, Adolescent Medicine and Neonatology, Faculty of Medicine, Medical Centre-University of Freiburg, 79106 Freiburg, Germany

**Keywords:** long-chain fatty acid oxidation disorders, LCHADD, lipidomics, sphyngomyelines and ceramides, neurodegeneration

## Abstract

Long-chain 3-hydroxyacyl-CoA deficiency (LCHADD) and mitochondrial trifunctional protein (MTPD) belong to a group of inherited metabolic diseases affecting the degradation of long-chain chain fatty acids. During metabolic decompensation the incomplete degradation of fatty acids results in life-threatening episodes, coma and death. Despite fast identification at neonatal screening, LCHADD/MTPD present with progressive neurodegenerative symptoms originally attributed to the accumulation of toxic hydroxyl acylcarnitines and energy deficiency. Recently, it has been shown that LCHADD human fibroblasts display a disease-specific alteration of complex lipids. Accumulating fatty acids, due to defective β-oxidation, contribute to a remodeling of several lipid classes including mitochondrial cardiolipins and sphingolipids. In the last years the face of LCHADD/MTPD has changed. The reported dysregulation of complex lipids other than the simple acylcarnitines represents a novel aspect of disease development. Indeed, aberrant lipid profiles have already been associated with other neurodegenerative diseases such as Parkinson’s Disease, Alzheimer’s Disease, amyotrophic lateral sclerosis and retinopathy. Today, the physiopathology that underlies the development of the progressive neuropathic symptoms in LCHADD/MTPD is not fully understood. Here, we hypothesize an alternative disease-causing mechanism that contemplates the interaction of several factors that acting in concert contribute to the heterogeneous clinical phenotype.

## 1. Introduction

The mitochondrial trifunctional protein (MTP) of the β-oxidation pathway is a α2β2 heterotetramer multienzyme complex encoded by two different genes, *HADHA* (OMIM 600890) and *HADHB* (OMIM 143450). This enzyme complex catalyzes three reactions. The long-chain enoyl-CoA hydratase and long-chain 3-hydroxyacyl-CoA dehydrogenase (LCHAD) are carried by the α-subunit, while the long-chain 3-ketoacyl-CoA thiolase (LCKAT) is localized on the β-subunit [1,2]. Mutations in *HADHA* are responsible for LCHAD deficiency (LCHADD), whereas variants in *HADHA* and *HADHB* cause MTP deficiency (MTPD). Both diseases are autosomal recessive disorders of the mitochondrial long-chain fatty acid oxidation (lc-FAODs) affecting the degradation of chain fatty acids with a chain length > C12 [3]. These diseases, with an incidence of 1:250,000 newborns in Germany, are characterized by a wide range of clinical manifestations including cardiomyopathy, liver dysfunction, progressive neuropathy and retinopathy, rhabdomyolysis and sudden death (https://www.screening-dgns.de/Pdf/Screeningreports/DGNS-Screeningreport-d_2019.pdf; accessed on 15 May 2022) [3]. The incomplete oxidation of long chain fatty acids during acute episodes of metabolic decompensation results in the accumulation of toxic acylcarnitines and energy deficiency. The subsequent impaired production of redox equivalents for oxidative phosphorylation may lead to life-threatening episodes, coma and death. Lc-FAODs are included in newborn screening programs of many countries worldwide. The identification of affected patients at neonatal screenings allows therapy to be initiated, thereby avoiding catabolic crises and metabolic derangement. No cure for LCHADD and MTPD is available. However, a number of dietary-based management strategies can be applied. Treatment recommendations include frequent meals, avoiding fasting, restriction of dietary long-chain fatty acids and supplementation with carbohydrates and medium-chain fatty acids (MCT oil) [4,5]. In addition, the use of triheptanoin, a triglyceride with three molecules of heptanoic acid, was recently approved by the FDA for the treatment of lc-FAODs [6].

Despite early diagnosis and therapy initiation, LCHADD and MTPD present with retinopathy and neurodegenerative symptoms originally attributed to the accumulation of toxic acylcarnitines, oxidative stress and energy deficiency. These symptoms specific for LCHADD and MTPD are progressive and cannot be avoided. Studies in the fibroblasts of patients with lc-FAODs have demonstrated that the accumulating fatty acids due to defective β-oxidation are likely involved in remodeling processes leading to an alteration of the profile and content of complex lipids [7,8]. 

Here, we aim at outlining the importance of these complex molecules in the pathophysiology of lc-FAODs. We summarize the evidence that exists in support of the hypothesis that profile disturbances of mitochondrial cardiolipins and sphingolipids are likely involved in LCHADD/MTPD disease development. Based on these observations, we propose a novel mechanism that considers dysregulated complex lipids as a contributing cause in the progressive neurodegenerative symptoms of LCHADD/MTPD. 

## 2. Mitochondrial Trifunctional Protein (MTP) and Cardiolipins

Very recently the crystal structure of the human MTP has been elucidated [2]. Due to the peculiar arcuate shape of the protein that binds its concave site to the mitochondrial inner membrane, the authors suggested a structure acting as a channel that allows the transition of the substrate among the catalytic units [2]. In particular, the transfer of the shortening acyl-residues from the hydratase to the ketothiolase is possible because of the hydrophobic nature of the substrates. These are able to move among the mitochondrial inner membrane to reach the last catalytic site for the cleavage. Therefore, the integrity of the mitochondrial membrane is essential to ensure a proper enzyme function [2]. In addition to mutations that directly affect the catalytic domain of the enzymes [9,10,11,12], several other variants severely impact the structural stability of the protein with the subsequent loss of the interaction between α and β subunits [1,13,14,15,16]. Maintenance of mitochondrial processes is also ensured by an intact cardiolipin composition as an essential constituent of mitochondrial membranes. The essential role of the MTP α-subunit in mitochondrial cardiolipin profiling can be explained by the identical structure of this enzyme with the newly identified monolysocardiolipin acyltransferase 1 (MLCLAT1) [17]. It is therefore conceivable that sequence variants affecting MTP enzyme activity result in an altered cardiolipin profile [7]. At the same time, an aberrant cardiolipin profile reduces mitochondrial fusion and fission processes [18,19,20] and leads to the destabilization of mitochondrial supercomplexes and the dysregulation of energy metabolism [7,8]. Analysis in human fibroblasts has highlighted that the LCHADD induces cardiolipin remodeling with a subsequent profile alteration and incorporation of the non-metabolized C16 fatty acids into cardiolipin species [7]. In keeping with previous data [18,19,20], LCHADD cell lines have been shown to present with dysregulated mitochondrial dynamics accompanied by mitochondrial dysfunction [21]. However, profile alteration was also observed in other classes of bioactive lipids such as sphingolipids, ceramides and hexosylceramides. 

## 3. Sphingomyelins and Ceramides: New Co-Player for Neurodegeneration in LCHADD?

The aberrant metabolism of sphingolipids characterized, i.e., by the accumulation of specific species with the subsequent alteration of ceramide/sphingomyelin ratio, glucosylceramides, ceramides and glactolsylceramides [22] results in the loss of neurons and their axis [23] or in neuronal dysfunction [24,25]. The accumulation of these lipids in lysosomes is typical for Niemann–Pick disease, Gaucher’s disease, Farber’s disease and Krabbe’s disease, a group of inherited metabolic diseases called lysosomal storage disorders (LSDs). LSDs share the accumulation of sphingolipids and ceramides with neurodegenerative disorders such as Alzheimer’s disease (AD), Parkinson’s Disease (PD) and Multiple Sclerosis (MS) [26]. Ceramides and sphingolipids belong to the group of bioactive lipids acting as messengers and involved in a wide range of cellular functions. Both lipid classes contribute structurally to the bilayer of membrane lipids and lipid rafts, playing a striking role in inflammation, apoptosis, neuronal differentiation and synaptic transmission in neuronal–glial connections [27,28]. These lipid classes are essential for the membrane compartmentalization of transporters and receptors [29,30,31]. Therefore, any perturbation of their metabolism induces the remodeling of the plasma membrane with the subsequent alteration of cellular functions [28,32] and the possible development of several neurological diseases [33]. Studies in fibroblasts from LCHADD patients have shown for the first time the alteration of complex lipids, specifically sphingolipids and ceramides. This finding is indicative of an unexpected common feature between LSDs and FAODs. Long-chain fatty acids that cannot undergo mitochondrial β-oxidation, appear not only to accumulate as acylcarnitines, but also to participate in lipid remodeling processes. Bioactive compounds such as ceramides and sphingomyelins are directly involved in these processes [7,34] which may suggest the participation of aberrant lipid profiles in the onset of neurodegenerative symptoms in LCHADD. Because neural cells are not able to rely efficiently on mitochondrial β-oxidation to energy supply to a great extent [35], we may speculate that in LCHADD the accumulation of the toxic hydroxyacids could play a marginal role in these cells. Indeed, fatty acid degradation is associated with the generation of superoxides, high oxygen consumption and risk of hypoxia that limits the regeneration of ATP by mitochondria during fatty acid degradation [36,37,38]. 

In addition to MS and amyotrophic lateral sclerosis (ALS) [26], the role of the sphingolipid family in retina degenerative diseases has also been recently reviewed [39]. This phenotype is typical for sphingolipidoses, a group of diseases belonging to LSD presenting with early neurodegeneration, retinal vascular abnormalities, degeneration of ganglion cells and blindness [39,40,41,42,43]. In particular, alterations of the content and composition of ceramides associated with inflammation and apoptotic response [44] by acting directly on mitochondria lead to photoreceptor death [45,46,47] and retinal epithelium dystrophy [48,49]. Our findings demonstrated that fibroblasts from LCHADD patients showed a peculiar alteration of the ceramide/hexosylceramide and sphingomyelin/hexosylceramide ratios, similar to those reported for AD, PD and MS. These data are strongly suggestive of a redirection of the sphingomyelin metabolic flux towards the biosynthesis of hexosylceramides [7]. This results in the alteration of the lipid composition, without however the excessive accumulation of certain lipid species as occurs in LSDs [50]. In particular, the composition of sphingomyelin in LCHADD fibroblasts was shifted towards shorter chains. In addition, the total sphingomyelin content was remarkably reduced but accompanied by a parallel increase in hexosylceramides [7]. These sphingolipids belong specifically to the group of cerebrosides [51]. They are an essential structural component of cell membranes assembled in lipid rafts, serve to maintain the stability of myelin and contribute to the axonal growth of neurons as well as to the proper function of oligodendrocytes [52,53]. The upregulation of ceramide biosynthesis from sphingomyelin has been reported to specifically activate inflammatory processes in neurons and oligodendrocytes leading to cell death [54]. Studies in the plasma of patients with PD demonstrated that the total content of ceramides and hexosylceramides with fatty acid chains between 16 and 26 were significantly higher compared to healthy controls [55]. In a similar manner, the specific elevation of hexosylceramides has been described also for SM, ALS and macular degeneration [55,56,57] in response to oxidative stress, mitochondrial dysfunction [58], immune reactions and neuroinflammation [59]. Because these pathomechanisms are at least in part common to LCHADD/MTPD, it is conceivable to suppose that the disruption of the sphingolipid profiles may contribute to the onset of the specific neurodegenerative symptoms [7]. 

Although this information does not completely clarify the molecular mechanism, it clearly indicates that a multifactorial system contributes to the clinical feature in these diseases. In contrast to other fatty acid oxidation disorders, LCHADD/MTPD are characterized by the high accumulation of hydroxylated acylcarnitines [60]. Previous studies have shown that these compounds indeed cause oxidative stress in rat brains [61] and lipotoxycity in fatty livers during pregnancy [62]. In this context, oxidative stress has been demonstrated to mediate or amplify neuronal dysfunction and trigger neurodegeneration in AD, PD and ALS due to its effect on calcium homeostasis as well as on mitochondrial energy and respiration [63,64]. Although some points still remain obscure, it appears that oxidative stress may likely influence mitochondrial sphingolipid metabolism. The activity of sphingomyelinase can indeed be upregulated by oxidative stress enhancing the biosynthesis of bioactive metabolites such as ceramides and derivatives [65,66]. At the same time, ceramide and ceramide analogs impair mitochondrial respiration by inhibiting electron transport at complex III [67]. 

**Figure 1:**
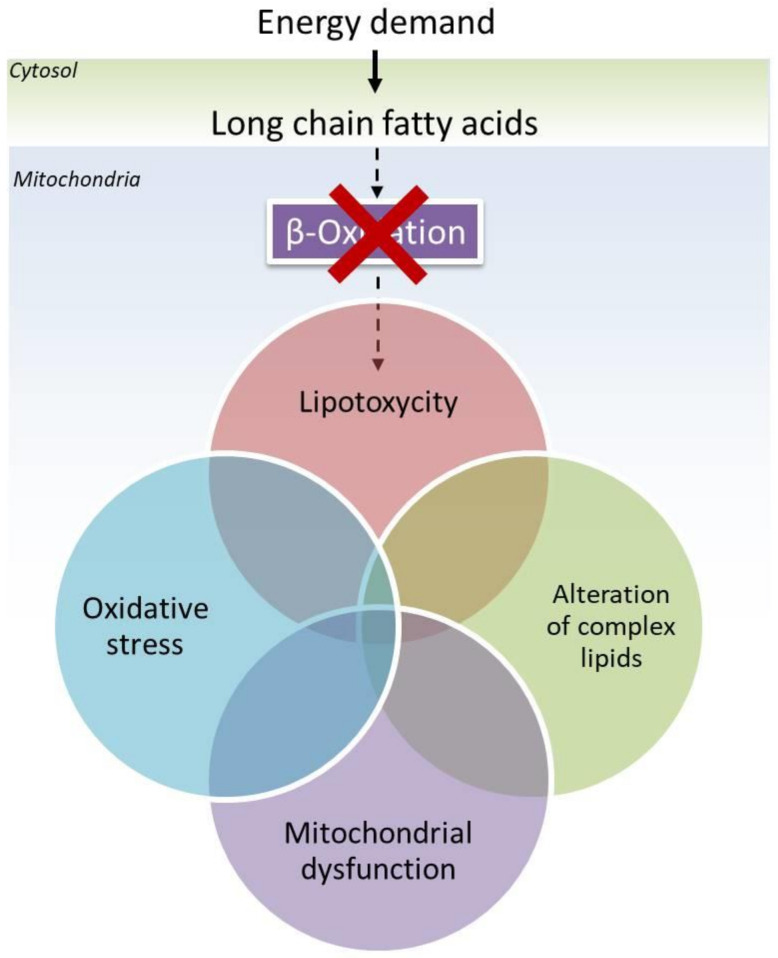
Schematic representation of the proposed working hypothesis on the additional involvement of complex lipids in the onset of neurodegenerative diseases in LCHADD/MTPD. It is known that due to impaired mitochondrial β-oxidation, cells accumulate toxic hydroxyl acylcarnitines. These organelles suffer oxidative stress that leads to mitochondrial dysfunction. At the same time an alteration of content and composition of complex lipids has been observed.

Based on these observations, here we propose a comprehensive pathomechanism for the progressive neurodegeneration in LCHADD/MTPD that includes the experimental evidence reported in the past years (Figure 1). First, oxidative stress induced by the accumulation of long-chain hydroxy acylcarnitines and hampered electron flow results in mitochondrial damage [68]. This is aggravated by the altered content and composition of mitochondrial cardiolipins as a consequence of the accumulation of long-chain fatty acids that cannot enter the β-oxidation cycle [7]. In this regard, it is known that altered cardiolipins affect mitochondrial respiration and dynamics and this effect has been recently described in fibroblasts from LCHADD patients [7,69]. However, cardiolipins are not the only lipid class that is subjected to remodeling. Indeed, sphingolipids change in content and profile and are characterized by an upregulation of the biosynthesis of hexosylceramides [8]. In particular, these lipids have been reported as detrimental for other neurodegenerative diseases [54]. In LCHADD/MTPD they may, therefore, share the co-responsibility for the specific development of retinopathy and peripheral neuropathy. This hypothesis is supported by the fact that the incubation of fibroblasts of LCHADD patients with medium chain fatty acids, especially heptanoate, avoids the biosynthesis and accumulation of long-chain hydroxy acylcarnitines, contributes to the restoration of the sphingolipid content and profile and makes this similar to those observed in the fibroblasts of healthy controls [8]. In accordance to this hypothesis, Francis et al. 2013 reported an improvement in oligodendrocyte survival and myelination in a mouse model of Canavan Disease [70]. The authors suggested that the energy supply via anaplerosis promoted myelination by reducing the metabolic demand placed on the oxidation of glucose by fatty acid synthesis [70]. It is therefore conceivable that this effect may also occur in other cells, thereby modulating the biosynthesis flux of complex lipids. However, studies to evaluate whether mitochondrial function is improved under these conditions are still ongoing and it is still unclear whether this effect may also occur in patients. 

## 4. Conclusions

In the light of the existing literature, it is clear that the dysregulation of complex lipids is a common element in the onset of several neurodegenerative diseases. In this context, the very recent reports on the altered composition of cardiolipins, sphyngomyelin and ceramides described in the fibroblasts of LCHADD patients led us to formulate the hypothesis of a more complex multifactorial pathogenesis behind the progressive neurodegenerative symptoms in this disease also involving the complex lipids. However, it is unclear whether the described alterations are a consequence of oxidative stress, remodeling or to the accumulation of fatty acids rather than an unforeseen independent effect. However, they may explain why despite therapy and metabolic maintenance, the symptoms remain progressive. 

We would also highlight the fact that these data have been retrieved from fibroblasts which of course never allow a full extrapolation to patients. Whether the same alteration of complex lipids and modulation by medium-chain fatty acids may also occur in vivo is currently under investigation. 

## Data Availability

The datasets supporting the conclusions of this article are included in the cited articles Alatibi et al. (2021) *Cells*. 18 May **2021**, *10*(5):1239 and Alatibi et al. (2021) *Int. J. Mol. Sci*. 29 September **2021**, *22*(19):10556.
